# Melatonin promotes the development of the secondary hair follicles by regulating circMPP5

**DOI:** 10.1186/s40104-023-00849-w

**Published:** 2023-04-07

**Authors:** Xiaogao Diao, Lingyun Yao, Tao Duan, Jiaxin Qin, Liwen He, Wei Zhang

**Affiliations:** Department of Animal Nutrition and Feed Science, State Key Laboratory of Animal Nutrition, College of Animal Science and Technology, China Agricultural University, Beijing, 100193 China

**Keywords:** Cashmere, MAPK, Melatonin, miR-211, Secondary hair follicle

## Abstract

**Background:**

The quality and yield of cashmere fibre are closely related to the differentiation and development of secondary hair follicles in the skin of cashmere goats. The higher the density of secondary hair follicles, the higher the quality and yield of cashmere from the fleece. Development of secondary hair follicles commences in the embryonic stage of life and is completed 6 months after birth. Preliminary experimental results from our laboratory showed that melatonin (MT) treatment of goat kids after their birth could increase the density of secondary hair follicles and, thus, improve the subsequent yield and quality of cashmere. These changes in the secondary hair follicles resulted from increases in levels of antioxidant and expression of anti-apoptotic protein, and from a reduction in apoptosis. The present study was conducted to explore the molecular mechanism of MT-induced secondary hair follicle differentiation and development by using whole-genome analysis.

**Results:**

MT had no adverse effect on the growth performance of cashmere kids but significantly improved the character of the secondary hair follicles and the quality of cashmere, and this dominant effect continued to the second year. Melatonin promotes the proliferation of secondary hair follicle cells at an early age. The formation of secondary hair follicles in the MT group was earlier than that in the control group in the second year. The genome-wide data results involved KEGG analysis of 1044 DEmRNAs, 91 DElncRNAs, 1054 DEcircRNAs, and 61 DEmiRNAs which revealed that the mitogen-activated protein kinase (MAPK) signaling pathway is involved in the development of secondary hair follicles, with key genes (*FGF2, FGF21, FGFR3, MAPK3 (ERK1)*) being up-regulated and expressed. We also found that the circMPP5 could sponged miR-211 and regulate the expression of *MAPK3*.

**Conclusions:**

We conclude that MT achieves its effects by regulating the MAPK pathway through the circMPP5 sponged the miR-211, regulating the expression of *MAPK3*, to induce the differentiation and proliferation of secondary hair follicle cells.

In addition there is up-regulation of expression of the anti-apoptotic protein causing reduced apoptosis of hair follicle cells. Collectively, these events increase the numbers of secondary hair follicles, thus improving the production of cashmere from these goats.

**Supplementary Information:**

The online version contains supplementary material available at 10.1186/s40104-023-00849-w.

## Introduction

**Fig. 1:**
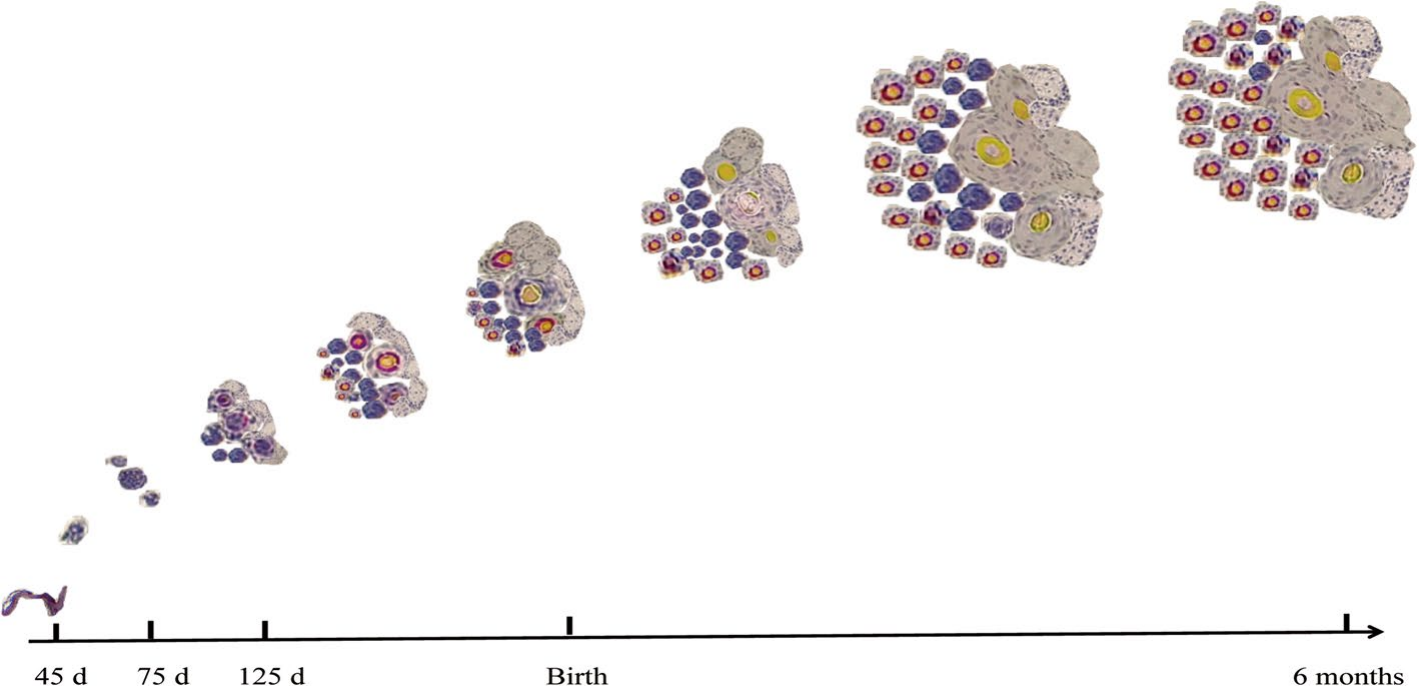
Proliferation and development of hair follicles in fetal and newborn cashmere goats. 45–75 d post conception: proliferation and development of primary hair follicles. 75–125 d post conception: adjacent structures appear; proliferation and development of secondary (cashmere) hair follicles, initiation of primary fibre production. 125 d post conception to birth: secondary hair follicles continue to proliferate; some cashmere fibre production. Birth to 6 months of age: the key period of secondary hair follicle growth

Cashmere fibres form the fleece of cashmere goats and produce a high-grade textile material with considerable economic value. Internationally, China, Mongolia and India are the main producers of cashmere [1]. The quality of cashmere is closely related to the diameter of individual fibres: the finer their diameter the higher the quality of the material. Hair follicles are subsidiary structures of the skin, categorised as either primary or secondary hair follicles. Primary hair follicles of cashmere goats produce coarse hair fibres of no commercial value, whereas secondary hair follicles produce the valuable cashmere fibres [2, 3]. Growth of cashmere fibre follows a seasonal pattern with growth from the secondary hair follicles commencing after the summer solstice but slowing down and eventually ceasing when daily photoperiods increase following the winter solstice. Differentiation and development of primary and secondary hair follicles commences during the embryonic stages of development of goats. The primary hair follicles are differentiated from epithelial cells, whereas secondary hair follicles branch off from the epidermal cell layer of the skin near the primary hair follicles. Primary hair follicles are fully developed at birth of the goats and the secondary hair follicles reach their full development at about 6 months of age (Fig. 1) [4, 5]. Formation of cashmere fibre ensues when the secondary hair follicles commence their cyclical pattern of activity [6]. For cashmere goats in Inner Mongolia, this means that formation of cashmere commences each year in July, stops growing from February to March in the following year, and is naturally shed from the skin by the end of April. Thereafter, from May to July, there is a non-growing cashmere period.

Melatonin (MT) is a neuroendocrine hormone involved in seasonal regulation of events such as the hair follicle growth cycle and has been applied to improve fur production in species such as fox, mink and rabbits [7–9]. MT has been used in goats to promote growth of cashmere fibre, but this has been complicated by alteration of the shedding process [10, 11]. Studies with administration of MT have been conducted by the cashmere goat team at China Agricultural University for nearly 20 years. They have used MT on adult cashmere goats successfully to: induce growth of secondary hair follicles, advance the onset of cashmere fibre growth, reduce the non-growing cashmere period, solve the problem of cashmere shedding, and improve cashmere yield and quality [12]. The greater the density of secondary hair follicles and the finer the fibre diameter, the better the yield and quality of the cashmere fleece. For goat kids, the period from birth to 6 months of age is critical for development of secondary hair follicles, and the number of fixed secondary hair follicles produced during this time determines the final number of secondary hair follicles, and thus the magnitude of the cashmere yield in adulthood. Preliminary results from our laboratory showed that treatment of young goats with MT shortly after birth: enhanced antioxidant levels in blood and skin, increased expression of the anti-apoptotic protein, and reduced apoptosis of hair follicle cells, thus increasing the number of secondary hair follicles and improving the quality and yield of cashmere [13]. We propose that MT increases the number of secondary hair follicles in goat kids in two ways: by promoting differentiation and proliferation of secondary hair follicle cells and by reducing the occurrence of apoptosis in these cells.

With the development of genomics, more studies show that non-coding RNA plays an important role in the development of hair follicle, and miRNA/lncRNA can directly or indirectly regulate the occurrence of hair follicle through targeted signaling pathways [14, 15]. However, there are few studies on circRNA in hair follicle development. Reports show that circRNA has a sponge effect on miRNA, and then regulates the expression of target genes [16, 17]. Therefore, based on the previous research, this study used genome-wide technology to explore the expression of non-coding genes and coding genes under MT intervention, and revealed the potential signaling pathways in which MT may be involved in secondary hair follicle growth and development. In addition, we developed corresponding tracking plans to study the long-term effects of MT on the growth of hair follicles and production of cashmere fibre in these cashmere goats.

## Materials and methods

### Animals and management

Thirty-two newborn cashmere goat kids with similar live weights (mean 3.00 ± 0.2 kg) were randomly allocated to two groups (*n* = 16); melatonin (MT) and control. Goats in the MT group were implanted s.c. with melatonin pellets to provide a dose of 2 mg/kg on d 1 from birth and at 2 and 4 months of age. The sustained release period of these pellets lasts for 60 d and the dosage is based on results of our previous studies [13]. Goats in the control group did not receive any treatment. From birth the goat kids were suckled by their dams that had lucerne and water freely available with supplementary starter feed (200 g/d, see Additional file 1: Table S1) provided as required. This study was conducted on the Yiwei white cashmere goat breeding farm at Ordos, Inner Mongolia, China (39°11′ N,107°16′ E).

### Live weight and collection of cashmere and skin samples

Live weight of the animals was recorded at birth, during the MT controlled-release period at 3 and 6 months of age, and at 1 and 2 years of age. Cashmere was harvested by combing at 3 and 6 months of age and after the first and second year of growth. Measurements of cashmere performances were conducted as described by Duan et al. [12]. Quadruplicate samples of skin were collected from the upper flank and right shoulder at 1 day of age, at 2, 4, and 6 months of age, and in the second year. Duplicate samples were placed immediately in liquid nitrogen for subsequent storage at −80 ℃ until they were subjected to transcriptome and biomolecular analyses. Microscopy with Sacpic staining was employed with the other duplicate samples to examine the morphology of hair follicles and to count their numbers. The main indices determined from the microscopic examination using procedures described by Yang et al. [13].

### Immunofluorescence analysis

Paraffin sections of skin samples underwent antigen repair using EDTA antigen repair buffer (pH 8.0) after deparaffinization and rehydration. Blocking was performed with hydrogen peroxide and rabbit serum, the corresponding primary and secondary antibodies were added (Ki-67—an indicator of mitotic activity (1:100, Abcam, Cambridge, UK), K-14—a keratin (1:500, Covance, Princeton, NJ, USA), Fn1 (1:500, Servicebio, Wuhan, China), Wnt-10a (1:500, Sanying, Wuhan, China)), then DAPI dye solution was added for DAPI re-staining of cell nuclei, and the preparations were sealed. Fluorescence of the stained sections was analyzed with a TCS SPE confocal microscope (Leica Microsystems, Bannockburn, IL, USA).

### Transcriptome analysis

The skin samples collected at 4 months of age underwent high-throughput sequencing analysis. RNA isolation, library construction, RNA sequencing, and analysis accomplished by Majorbio Co., Ltd. (Shanghai, China). The Majorbio cloud platform and software were used for analysis the items according to relevant literature and instructions [18–22].

### RT-qPCR and western blotting

The RT-qPCR was performed using a Fluorescence Quantitative PCR Kit (Takara Bio, Kusatsu, Shiga, Japan). For analysis of RNA expression, RT-qPCR was carried out using the primer (Tsingke Biotechnology Co., Ltd., Beijing, China) according to the manufacturer’s instructions. Levels of mRNA, microRNA and circulating RNA expression were calculated by 2^−ΔΔCt^ method. The proteins extracted from skin tissue blocks were quantified using the bicinchoninic acid method (BCA protein assay) and adjusted to the same concentration as the candidate proteins. Proteins were loaded onto the SDS-PAGE gel, electrophoresed, and analyzed by Western blotting using antibodies against the protein under test. The Western blotting results were visualized using a high-sensitivity chemiluminescence detection kit (Beyotime, Shanghai, China), and the X-ray film was exposed for developing and photographing.

### Luciferase gene reporter assay

Primers for amplifying target genes and target gene 3′-untranslated regions (UTRs) were based on gene sequences in Gen Bank, and 3′-UTR sequences were amplified by PCR using cashmere goat genomic DNA as template. PCR products were cloned into the dual-luciferase reporter gene vector to construct the wild-type plasmid. The target sequence of chi-miR-211 in the mitogen-activated protein kinase (*MAPK3*) gene was mutated to construct mutant plasmids. Finally, expression of the luciferase reporter was measured, and the target sites of miRNAs in the transfected 3′-UTRs were analyzed. The plasmid and chi-miR-211 mimics were synthesized by Hanheng Biotechnology Co., Ltd. (Shanghai, China). For the circRNA test, 293 T cells were co-transfected with wild type (WT) or mutant circMPP5 and miR-211-mimics or negative control-mimics (NC-mimics) using Lipofectamine 3000. Renilla luciferase activity was normalized to Firefly luciferase. After transfection for 48 h, cells were subjected to dual-luciferase analysis.

### Statistical analyses

The SPSS27.0 and the GraphPad 9.0 were employed in this study. Results were expressed as mean value ± SD. Data on the cashmere performances and parameters related to hair follicle by Student’s *t*-test. Difference was considered significant at *P* < 0.05.

## Results

### Growth performance

**Fig. 2:**
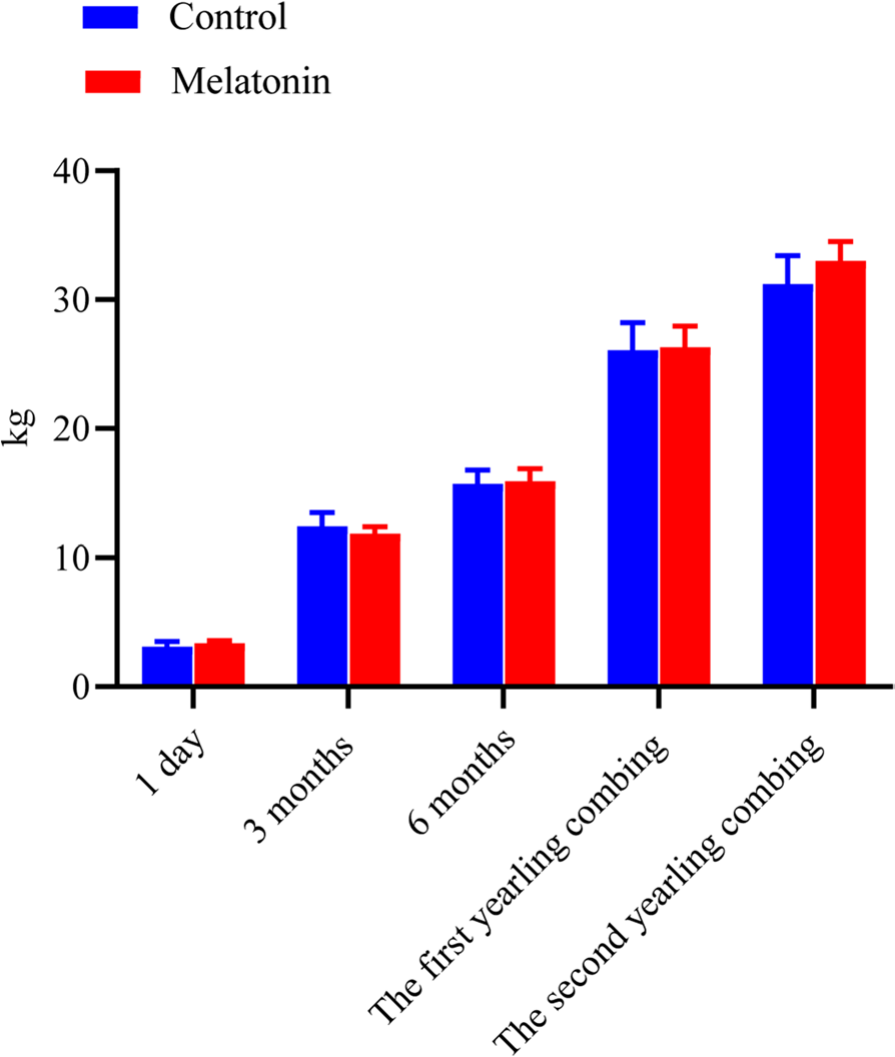
Effects of melatonin implantation on live weight of goat kids from birth to 2 years of age

There was no effect of the melatonin treatment on live weight at any of the times the animals were weighed (Fig. 2). At the end of the MT treatment period (6 months of age) mean live weights were 15.93 ± 0.96 and 15.70 ± 1.08 kg for treated and control groups, respectively (*P* > 0.05, Fig. 2).

### Cashmere production

**Fig. 3:**
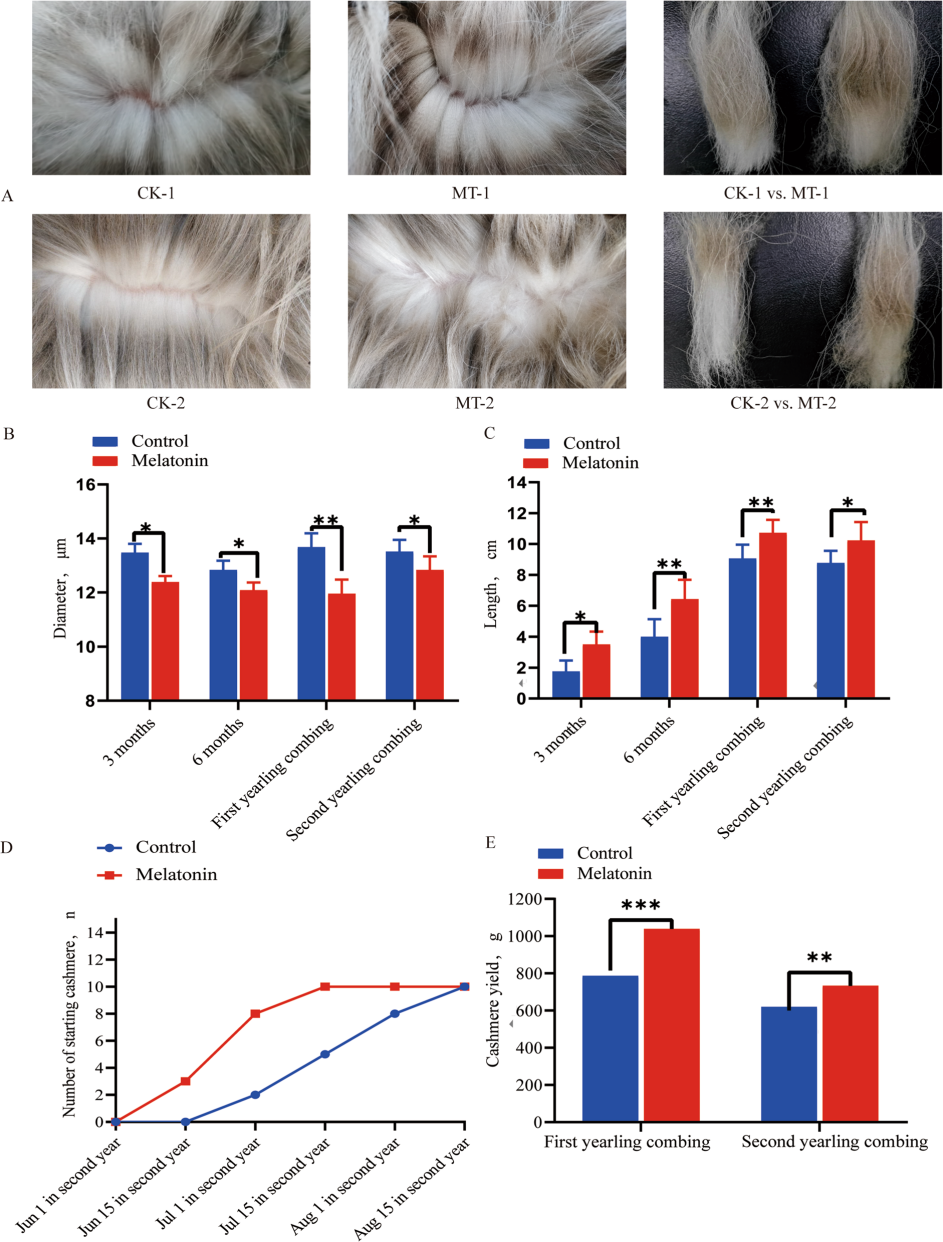
Effects of melatonin on cashmere growth. **A **Photographs of selected animal’s fleeces (CK—control, MT—melatonin treated) at 1^st^ (1) and 2^nd^ (2) combing. **B** Cashmere fibre diameter of MT-treated and control goats at different times following treatment. **C** Cashmere fibre length of MT-treated and control goats at different times following treatment. **D** Number of control and MT-treated goats initiating fibre growth at different times during the second year following treatment. **E** Cashmere yield of MT-treated and control goats at each of the first 2 years following treatment. Values represent means ± SD. ^*^P < 0.05, ^**^P < 0.01, ^***^P < 0.001 MT-treated vs. controls

Treatment of the goat kids with MT generated an improvement in cashmere growth that was visible in relation to that in the control animals (Fig. 3A). Following treatment with MT, cashmere fibre diameter was reduced by 1.73 μm at the first year (*P* < 0.01) and by 0.68 μm in the second year (*P* < 0.05) (Fig. 3B). The staple length was increased on all four occasions and differing by 1.46 cm in the second year (*P* < 0.05) (Fig. 3C). The pattern of onset of cashmere fibre growth, which was monitored by counting the numbers of goats with growing fleeces throughout the second year after the MT treatment (Fig. 3D), showed an advance of onset of about one month in the treated goats. As well as effects on fibre quality and onset of growth, MT increased the yield of cashmere in both years following treatment, by about 252 g in the first year’s combing (*P* < 0.01) (Fig. 3E). The increase was about 115 g in the second year (*P* < 0.05), when the overall yield was lower (Fig. 3E).

### Primary and secondary hair follicles

**Fig. 4:**
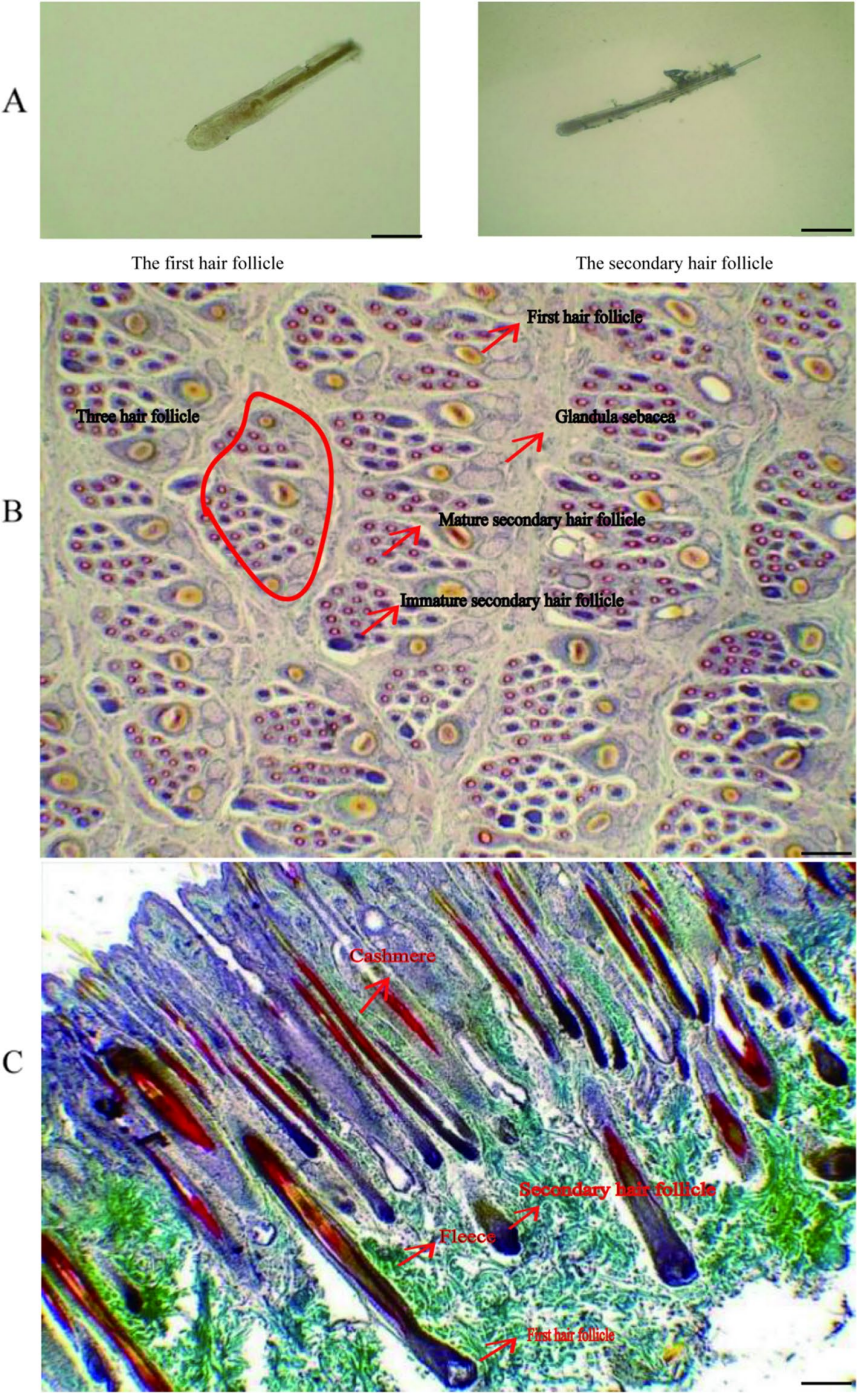
Photomicrographs of skin sections showing primary and secondary hair follicles. **A** Unstained primary and secondary hair follicles. **B** Sacpic stainining transverse section. **C** Sacpic staining longitudinal section. Scale bar = 200 μm

**Fig. 5:**
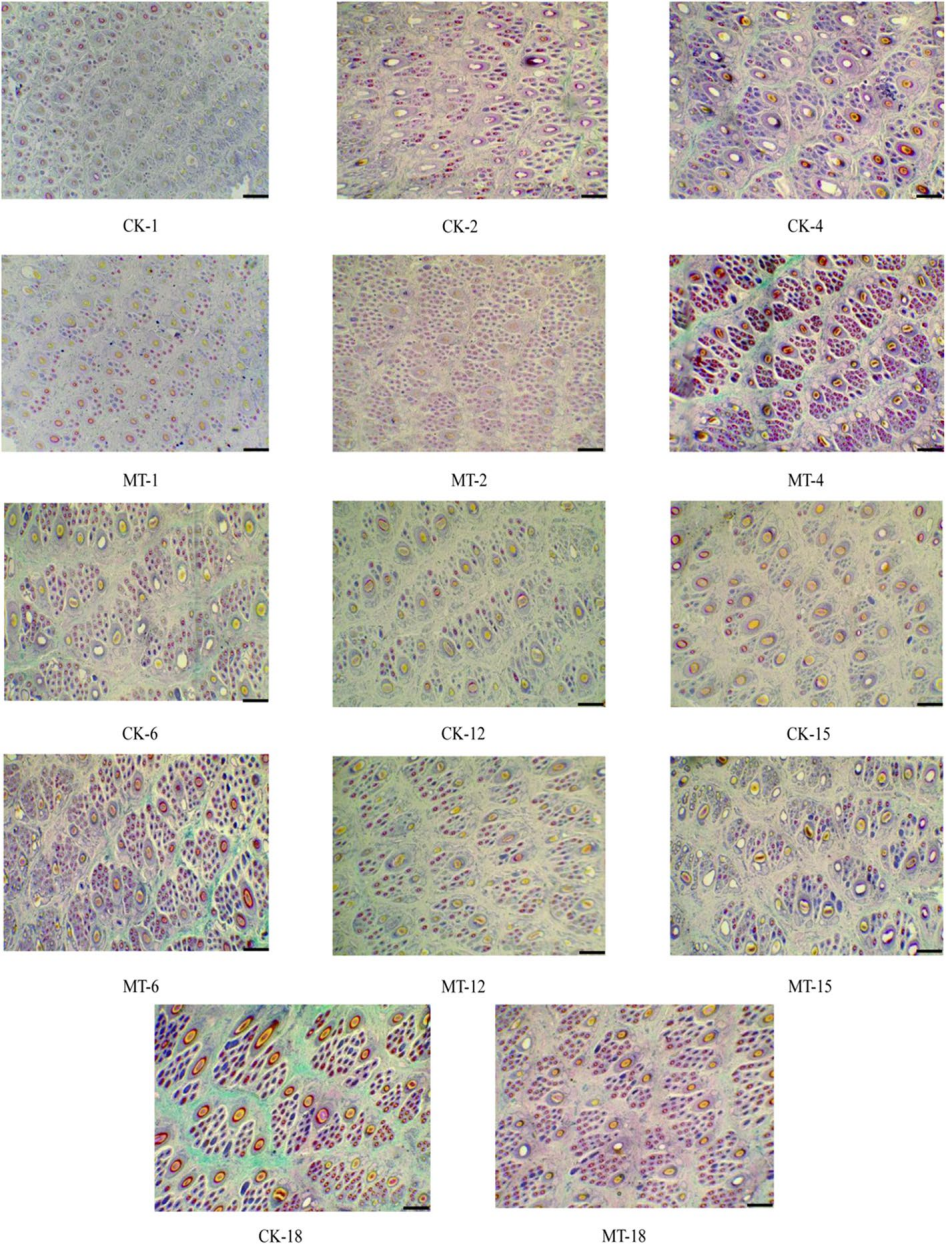
Representative photomicrographs (40 × , Sacpic staining) showing transverse sections of skin of MT-treated (MT) and control (CK) goats taken from 1 to 18 months of age. The numbers represent months of age, Scale bar = 200 μm

**Fig. 6:**
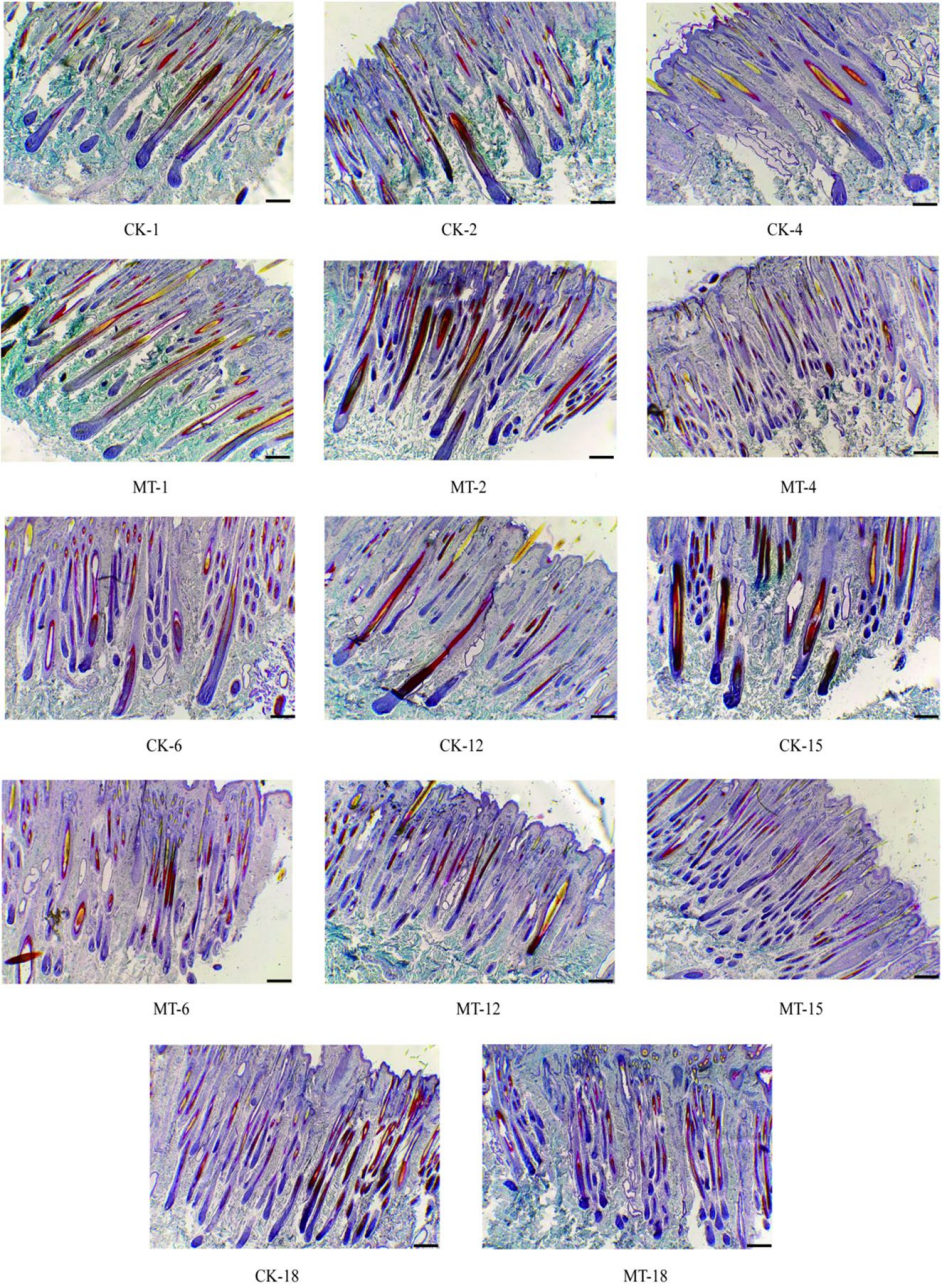
Representative photomicrographs (40 × , Sacpic staining) showing longitudinal sections of skin of MT-treated (MT) and control (CK) goats taken from 1 to 18 months of age. The numbers represent months of age, Scale bar = 200 μm

**Fig. 7:**
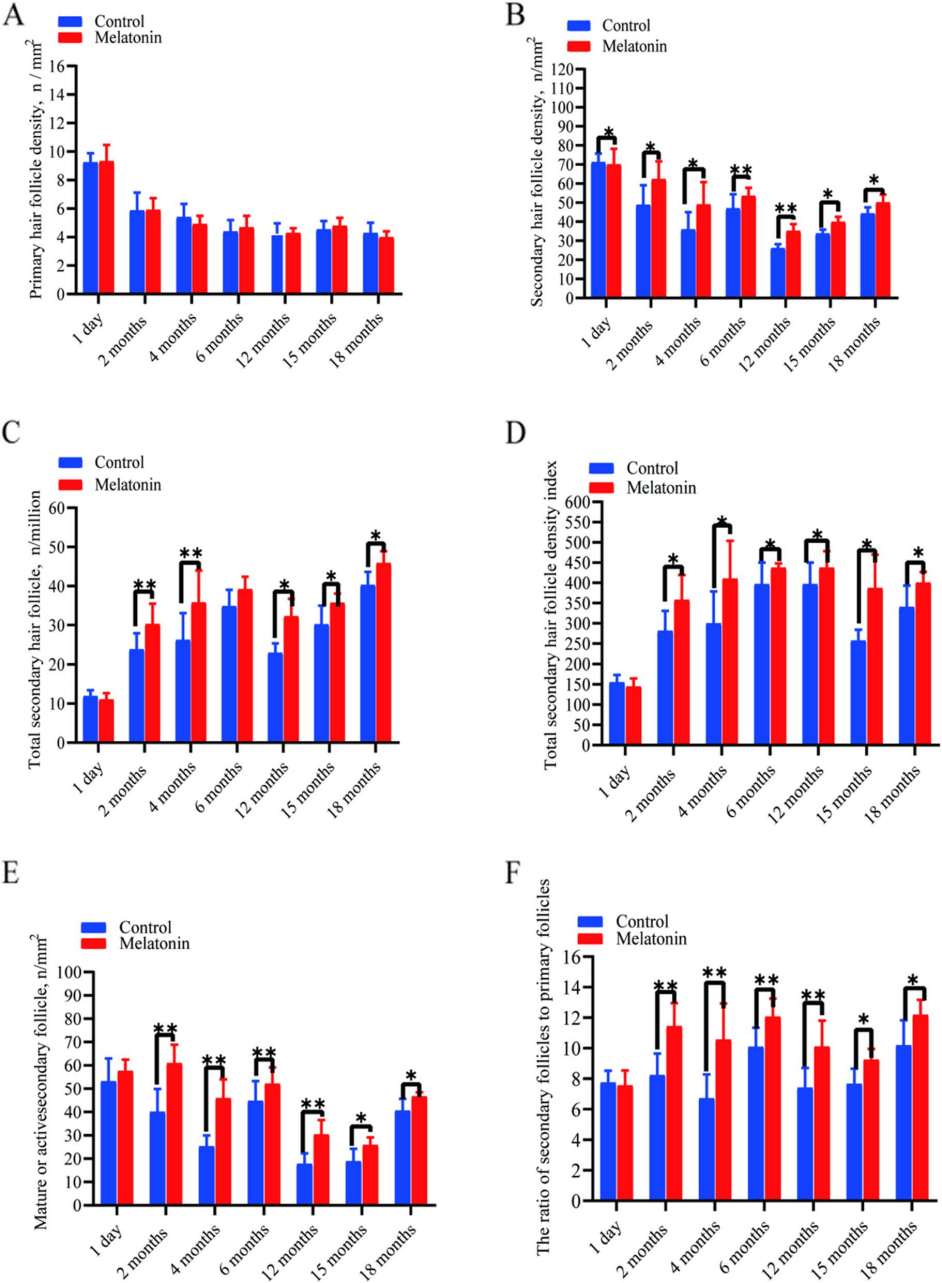
Hair follicle density, activity and ratio of secondary:primary hair follicles of MT-treated and control goats recorded from birth to 18 months of age. **A** Primary hair follicle density. **B** Secondary hair follicle density. **C** Secondary hair follicle total number. **D** Secondary hair follicle density index. **E** Mature or active hair follicle density. **F** The ratio (S:P) of secondary hair follicles to primary hair follicles.Values represent means ± SD. Different symbols (*, **) above the bars indicate level of significance of differences (MT-treated vs. controls, ^*^P < 0.05, ^**^P < 0.01)

Representative photomicrographs of hair follicles are provided in Fig. 4. Consecutive representative photomicrographs from skins of MT-treated and control goats taken at each of the seven sampling stages are shown in Fig. 5 and 6. These figures provide visual impressions of the effects of MT treatment that are summarized graphically in Fig. 7. In the case of primary skin follicle density, there was no difference between treated and controls at any stage (Fig. 7A), the value being higher at 1 day of age for both groups than thereafter. However, in the case of secondary skin follicles, MT treatment of the goats led to improvements in the parameters recorded here (Fig. 7B–F). Apart from the day when the MT implants were applied (day 1 of age), MT treatment increased the measures of density of secondary skin follicles at almost all other sampling times (Fig. 7B–E). The ratio of secondary to primary skin follicles was increased invariably by treatment with MT (Fig. 7F).

### Proliferation of hair follicle cells

**Fig. 8:**
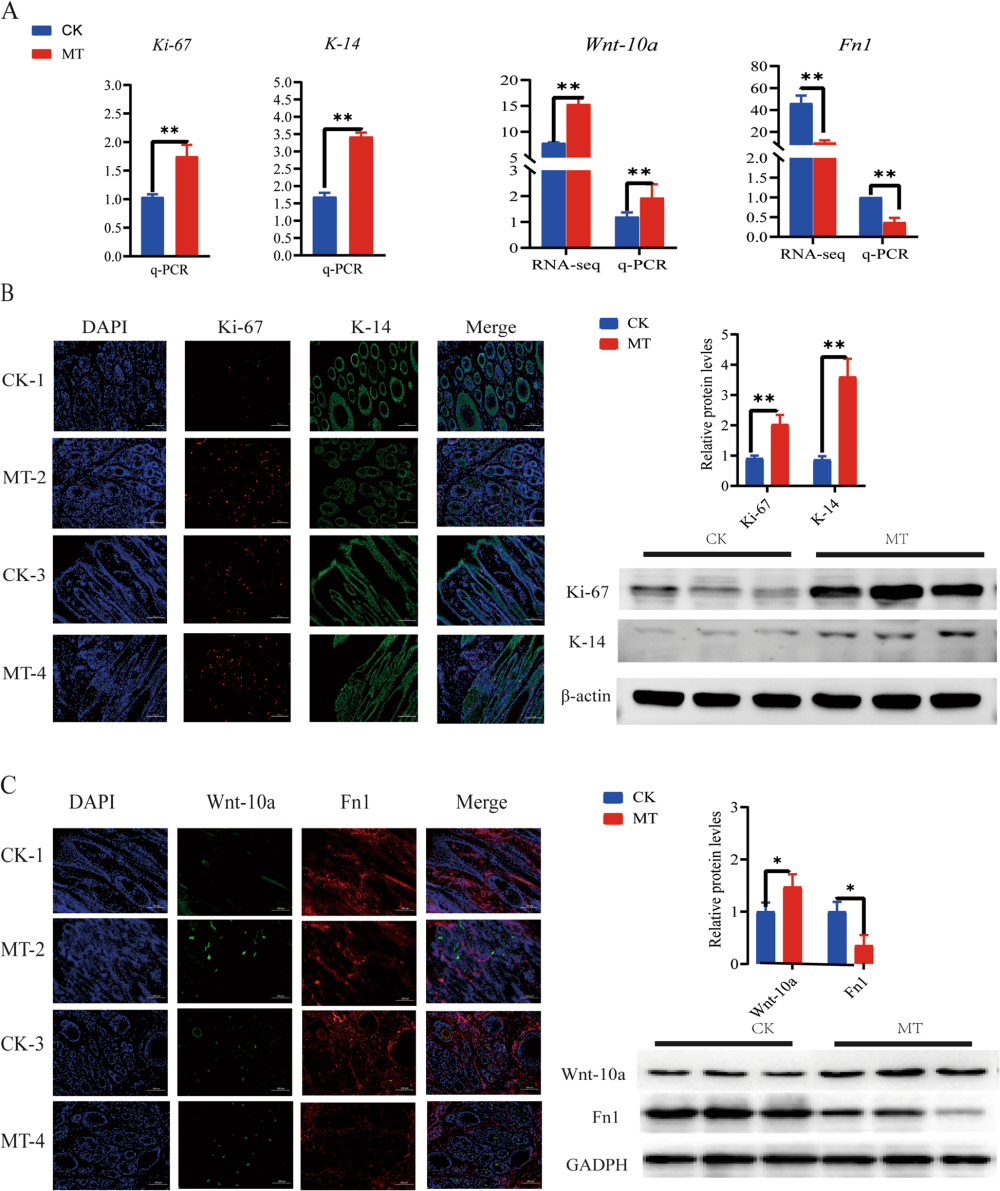
Melatonin promoting the proliferation and differentiation of secondary hair follicle cells. **A** The q-PCR and RNA-seq of *Ki-67*, *K-14, Wnt-10a* and *Fn1*. **B** and **C** The fluorescence signal DAPI (blue), Ki-67 and Fn1 (red), K-14 and Wnt-10a (green), merge in the transection ( CK-1 and MT-2) and longitudinal section ( CK-3 and MT-4), Scale bar = 100 μm, and another is WB. ^*^P < 0.05, ^**^P < 0.01

The results of *Ki-67, K-14, Wnt-10a* and *Fn1* genes in the CK groups showed significant differences. Based on these gene results, protein expression was analyzed. The optical signal of immunofluorescence arising from *Ki-67*, an indicator of mitotic activity, was much stronger in paraffin sections from skin of the MT-treated animals than in those from controls. Likewise, the keratin, *K-14*, produced a stronger signal in MT-treated animals indicating enhanced cell differentiation in this group, and similar result were observed for *Wnt-10a*, while *Fn1* shows the opposite result. The results of western blot (WB) also verified the protein expression again (Fig. 8B, C).

### MAPK signaling pathway

**Fig. 9:**
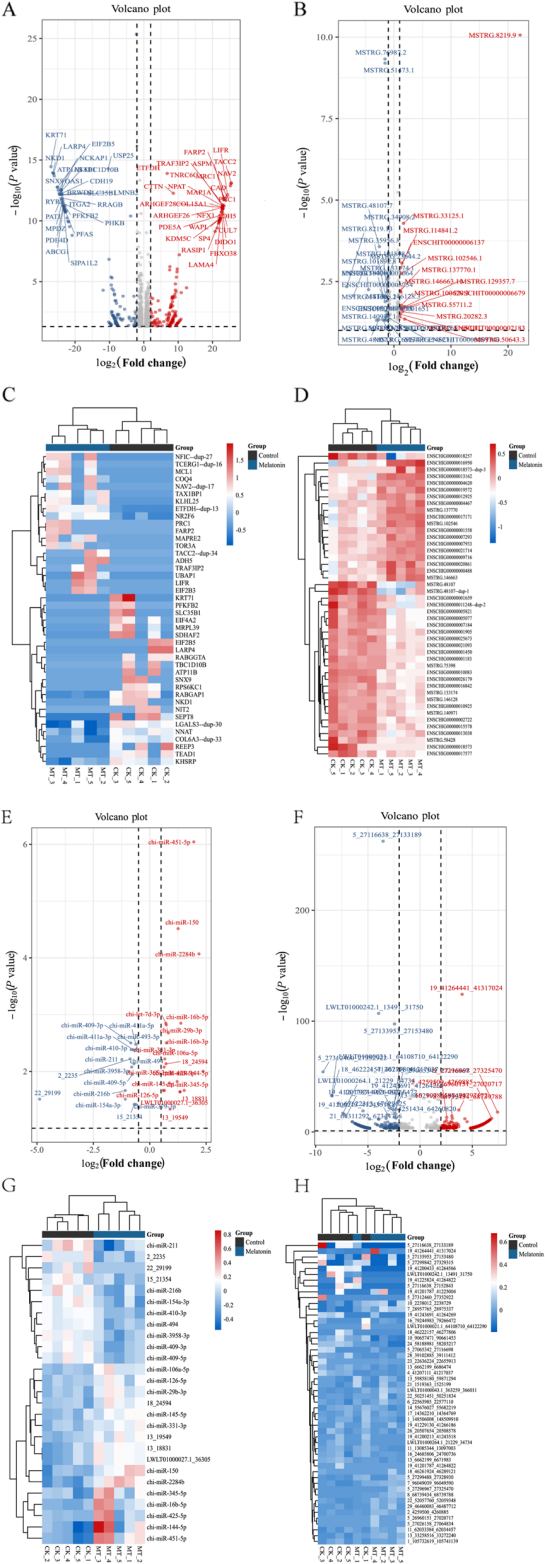
Differentially coding and non-coding gene expression in skin tissue activated by melatonin. **A** and **C** The volcano plot and heat map of DEmRNA. **B** and **D** The volcano plot and heat map of DElncRNA. **E** and **G** The volcano plot and heat map of DEmiRNA. **F** and **H** The volcano plot and heat map of DEcircRNA

**Fig. 10:**
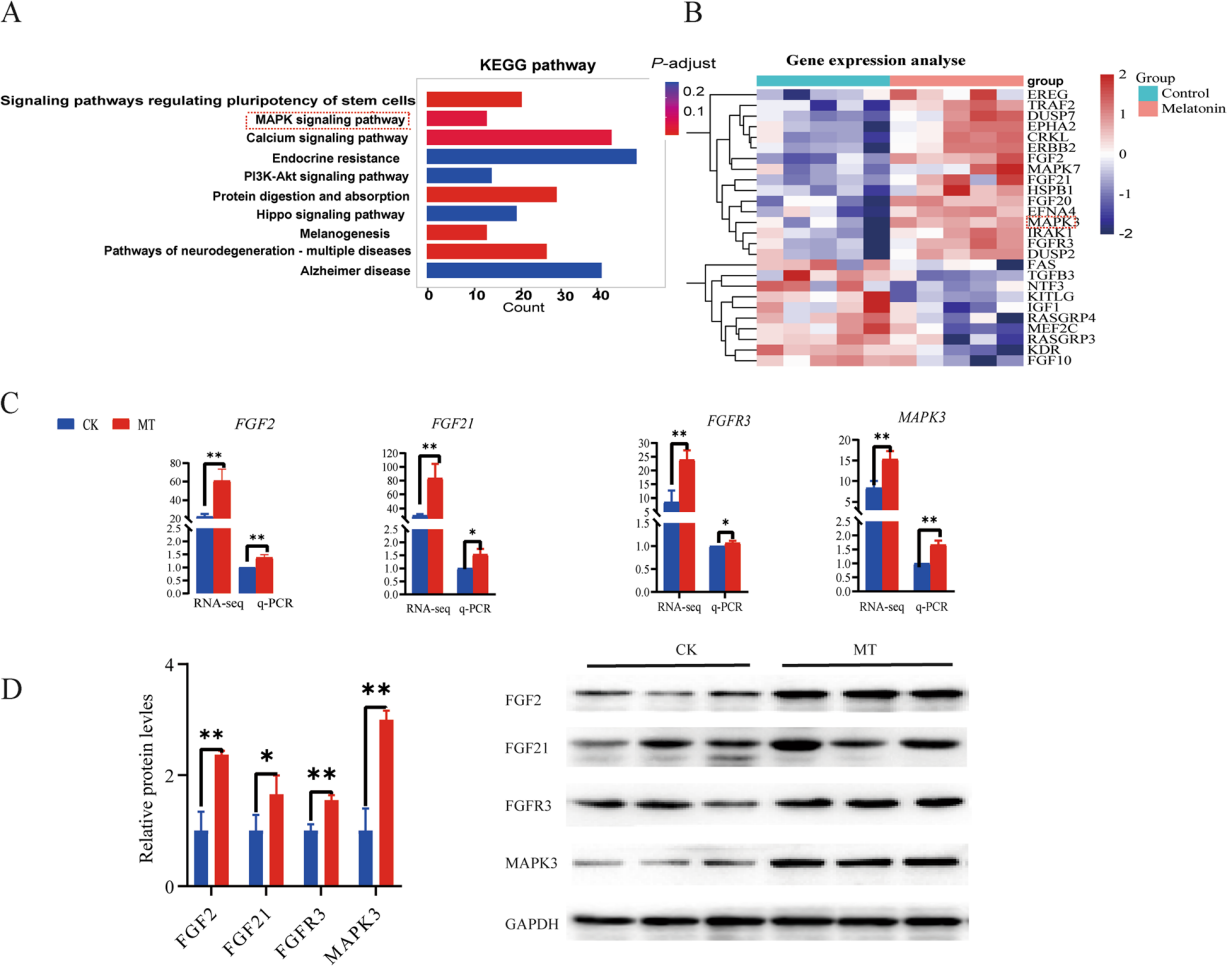
Upregulation of gene expression for proteins in the MAPK pathway by melatonin. **A** The KEGG analysis for the mRNAs. **B** The differential expression mRNA in MAPK signaling pathway. **C** The RNA-seq and q-PCR. **D** WB of key genes of MAPK signaling pathway. ^*^P < 0.05, ^**^P < 0.01

Data for the various RNAs investigated in this study are listed in Additional file 1: Table S2 and S3. The results showed that there were 1044 DEmRNAs (609 upregulation and 435 downregulation), 91 DElncRNAs (46 upregulation and 45 downregulation), 1054 DEcircRNAs (484 upregulation and 570 downregulation), and 61 DEmiRNAs (33 upregulation and 28 downregulation) in the control group compared with the MT group, respectively. The volcano plots and the heat maps for these are presented in Fig. 9. Using the KEGG enrichment analysis to determine the signaling pathways that involve the DEmRNAs, specific enrichment of genes was observed for signaling pathways including: regulating pluripotency of stem cells, MAPK signaling pathway, calcium signaling pathway, endocrine resistance, PI3k-Akt signaling pathway, protein digestion and absorption, hippo signaling pathway, melanogenesis, pathways of neurodegeneration-multiple diseases, Alzheimer’s disease (Fig. 10A). In the MAPK pathway, key genes such as fibroblast growth factor-2 (*FGF2*), *FGF21*, *FGFR3* and *MAPK3* were up-regulated (Fig. 10B). The results of q-PCR and western blotting (Fig. 10C and D) showed that the protein expression of several essential genes in the MAPK pathway in the MT group was higher than those in the controls.

**Fig. 11:**
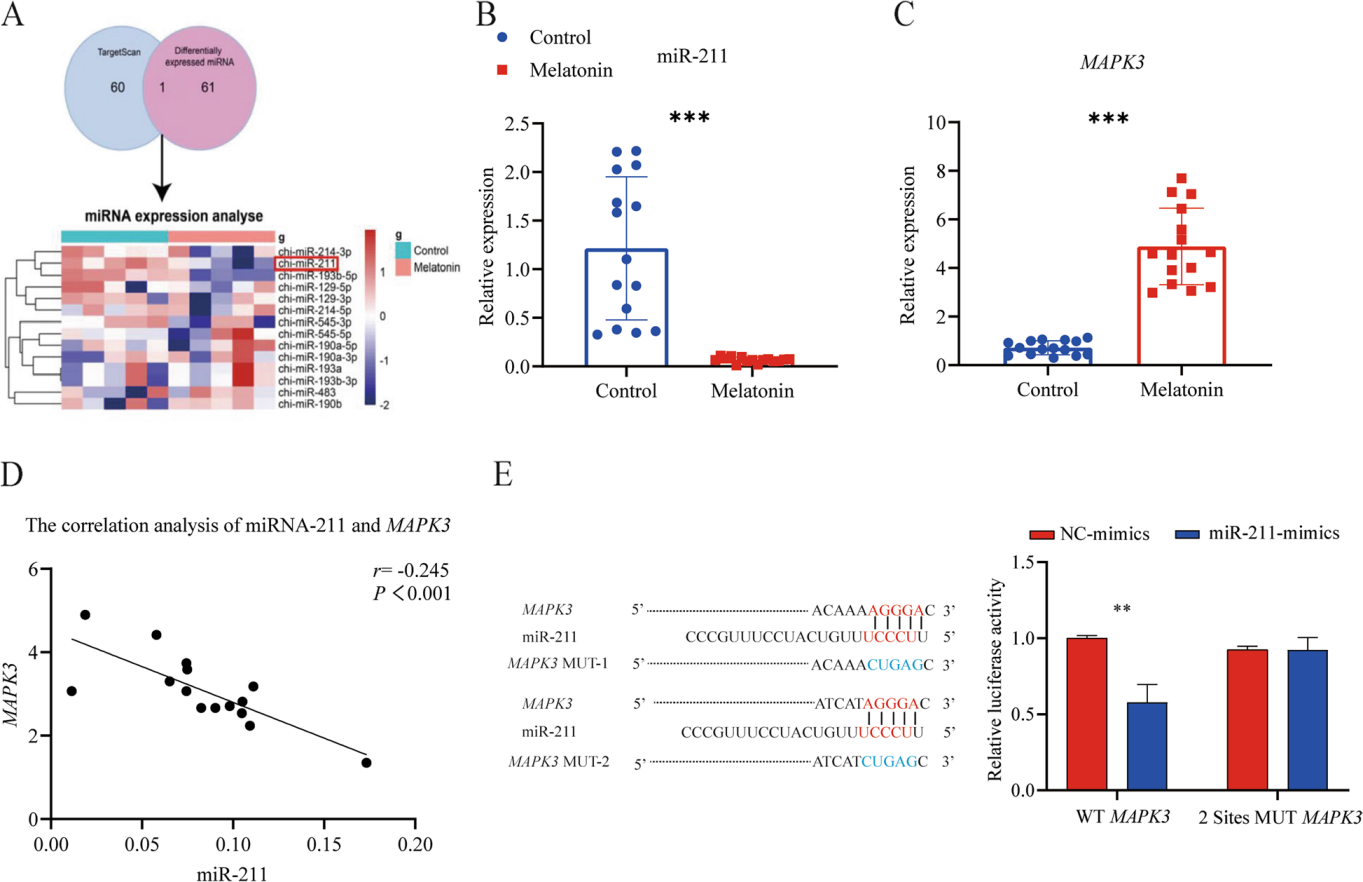
The miR-211 regulates the *MAPK3*. **A** Schematic illustration showing the target *MAPK3* of DEmiRNA from MT and CK group. **B** and **C** Quantitative real-time PCR assays of miR-211 and *MAPK3* in MT and control group. **D** Correlation analysis of miR-211 and *MAPK3* expression. **E** Predicted complementary binding sites between miR-211 and *MAPK3*, and verified by luciferase activity assay. ^**^P < 0.001, ^***^P < 0.01

**Fig. 12:**
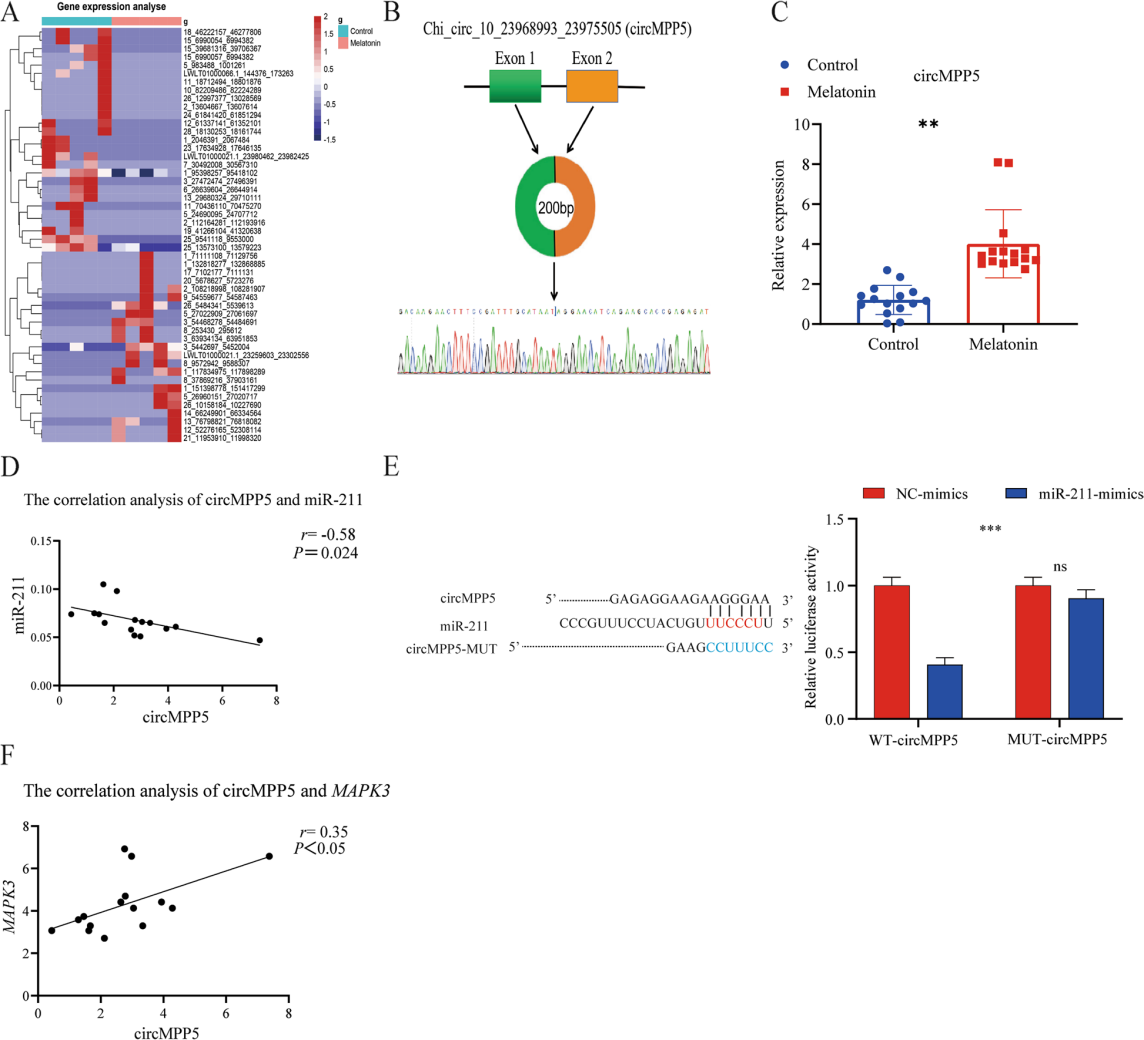
CircMPP5 serves as a sponge for miR-211. **A** Potential circRNAs that can regulate miR-211. **B** circMPP5. **C** Quantitative real-time PCR assays of circMPP5 in MT and control group. **D** Correlation analysis of circMPP5 and miR-211. **E** Predicted complementary binding sites between circMPP5 and miR-211, and verified by luciferase activity assay. **F** Correlation analysis of circMPP5 and *MAPK3* expression. ^**^P <0.01, ^***^P <0.001

*MAPK3*, a key gene in the MAPK pathway, proved to be a target gene of miR-211 (Fig. 11A). Levels of gene expression (determined from q-PCR) of miR-211 were higher for controls than for MT-treated goats whereas in the case of *MAPK3*, gene expression was greater in the MT group (Fig. 11B and C) and this accounts for the negative correlation between expression levels of these genes (Fig. 11D). Sequence alignment of miR-211 with *MAPK3* revealed that miR-211 possessed binding sites for *MAPK3-3*′-UTR, and *MAPK3* and these can be confirmed as potential target genes of miR-211. From the dual-luciferase reporter gene assay system, it was shown that miR-211 downregulated (*P* < 0.01) expression of the wild-type *MAPK3-3*′-UTR, indicating binding between the two molecules, and this downregulation effect disappeared after two mutations (*P* > 0.05), indicating that the mutation was successful (Fig. 11E). In addition, analysis of the DEcircRNA data to explore the interaction between circRNA and miR-211 under the intervention of MT showed that circRNAs such as circ_15_6990054_6994382, circMPP5, circ_5_26960151, circATE1 and circPI3R4, had targeting ability with miR-211 in the MT-treated animals (Fig. 12A, B). q-PCR data showed that levels of circRNA differed between MT-treated and control groups (Fig. 12C) and there was a negative correlation between circMPP5 and miR-211 (Fig. 12D). The dual luciferase reporter gene results showed that miR-211 reduced circMPP5 luciferase intensity in comparison with the mutated (Mut) vector (Fig. 12E). The circMPP5 was positively correlated with *MAPK3* (Fig. 12F)*.*

## Discussion

This study was conducted to investigate the potential mechanisms via which treatment of young goats with MT is able to promote the differentiation and development of secondary hair follicles in their skin. The results show that MT regulates the MAPK pathway in goats by activating the circMPP5/miR-211/MAPK3 to promote the differentiation and proliferation of secondary hair follicles, thereby improving the quality and yield of cashmere. Furthermore, this MT-treatment effect occurred in both the current and subsequent cashmere growing seasons indicating a potential enhancement of lifetime cashmere production from these goats.

As in other studies where MT has been used to enhance fibre production in goats and rabbits [23–25] the present results confirm this is achieved without effect on live weight or growth performance of the animals. As a textile fibre, the quality of cashmere is improved by reducing fibre diameter and increasing its length [26, 27] and the improvements in these parameters generated here by the treatment with MT has also been recorded in our earlier studies and by others [28, 29]. These effects of MT are achieved by advancing the onset of secondary follicle activity and by increasing the total number of active secondary hair follicles [30, 31] as can be deduced from the various indices of secondary follicle numbers and activity recorded here. Persistence of the fibre growth improvement into the second growth season following MT did not occur in our earlier study [32], so this finding may not be a consistent outcome of the treatment. Likewise, there is no published evidence of the treatment affecting fibre production by offspring of treated animals [33, 34].

Release of endogenous MT from the pineal gland of adult animals is regulated by changes in daily photoperiod [35] which explains why the naturally-occurring changes in fibre production are linked to seasons of the year. However, differentiation and development of hair follicles in goats begins before birth and is largely completed by about 6 months of age, prior to entering the annual hair growth cycle [8, 10, 36]. This explains why treatment of the goats with MT at birth is able to influence the subsequent cashmere growth cycles. Specific protein markers (biomarkers) of proliferation of secondary hair follicle cells such as keratin 14 (K-14) [37, 38] and Wnt10a [39–43] and their genes were elevated in the MT group whereas expression and protein levels of fibrinolectin (Fn1), an indicator of cell maturation [44–46], were lowered.

Whole transcriptome sequencing of extracts from the skin involving epithelial regions where actively growing hair follicles are located [47, 48] may provide markers for breed selection [49] and, importantly for the present study, can reveal gene expression activated by the MT treatment applied in this study, especially those relating to secondary hair follicles [50, 51]. The MAPK signaling pathway is one of the aggregation pathways of cellular information transmission induced by extracellular signals, including extracellular growth factor, extracellular growth factor receptor, and intracellular factors [52, 53]. As well as MT, insulin, platelet growth factor receptor and fibroblast growth factors (*FGFs*) can affect hair follicle growth by regulating the MAPK signaling pathway [54–56]. *FGF20* controls the formation of secondary hair follicles and there is a high expression of *FGF2, FGF21* and *FGF*-receptor genes during growth of secondary hair follicles [57, 58]. Extracellular signal-regulated kinases (ERKs, also known as MAP kinases) are vital proteins in the differentiation, proliferation and survival of epidermal stem cells [59–61]. In the present study, genes for the key extracellular and intracellular factors, *FGF2, FGF21, FGFR*, and *MAPK3 (ERK1)* in the MAPK pathway showed an up-regulation trend following MT treatment, providing strong evidence that MT exerts its influence on secondary hair follicles via effects on the MAPK pathway.

There is an increasing body of evidence for miRNA having a key role in the early development of hair follicles [62, 63]. *Ocu-miR-205* can promote hair follicles from the anagen to catagen stage by regulating the expression of genes for proteins in the notch and bone BMP signaling pathways [64]. Other studies showed that miRNA-203, miRNA-214 and miRNA-195-5p express the genes which regulate the development of hair follicles in cashmere goats [65, 66] and that MT can influence these miRNAs in the hair follicles of cashmere goats [67, 68]. miRNA-211 can regulate the MAPK3 pathway negatively, so it is in keeping with its role in this pathway that MT reduced miR-211 in the present study. CircRNA can compete with miRNA to regulate gene expression [69] and is a component of circRNA/miRNA/mRNA involved in regulatory networks as diverse as hepatocellular carcinoma (*HCC*), contraction of skeletal muscle [70] and proliferation of myoblasts [71]. Differentially expressed circRNAs have been studied in hair follicles of cashmere goats [72] and Angora rabbits [73]. The targeted binding relationship between miRNA-27b-3p and circRNA3236, miRNA-16b-3p and miR-16b-3p revealed that circRNA regulates gene expression by binding miRNA, thereby controlling hair follicle proliferation and fibre production in cashmere goats [74]. The present study adds to this body of knowledge by its examination of the differentially expressed circRNAs under the stimulatory influence of MT. The results show that MT in goats can competitively bind miR-211 through circMPP5 thus enhancing expression of the target gene *MAPK3* and promoting differentiation of secondary hair follicle cells.

## Conclusion

**Fig. 13:**
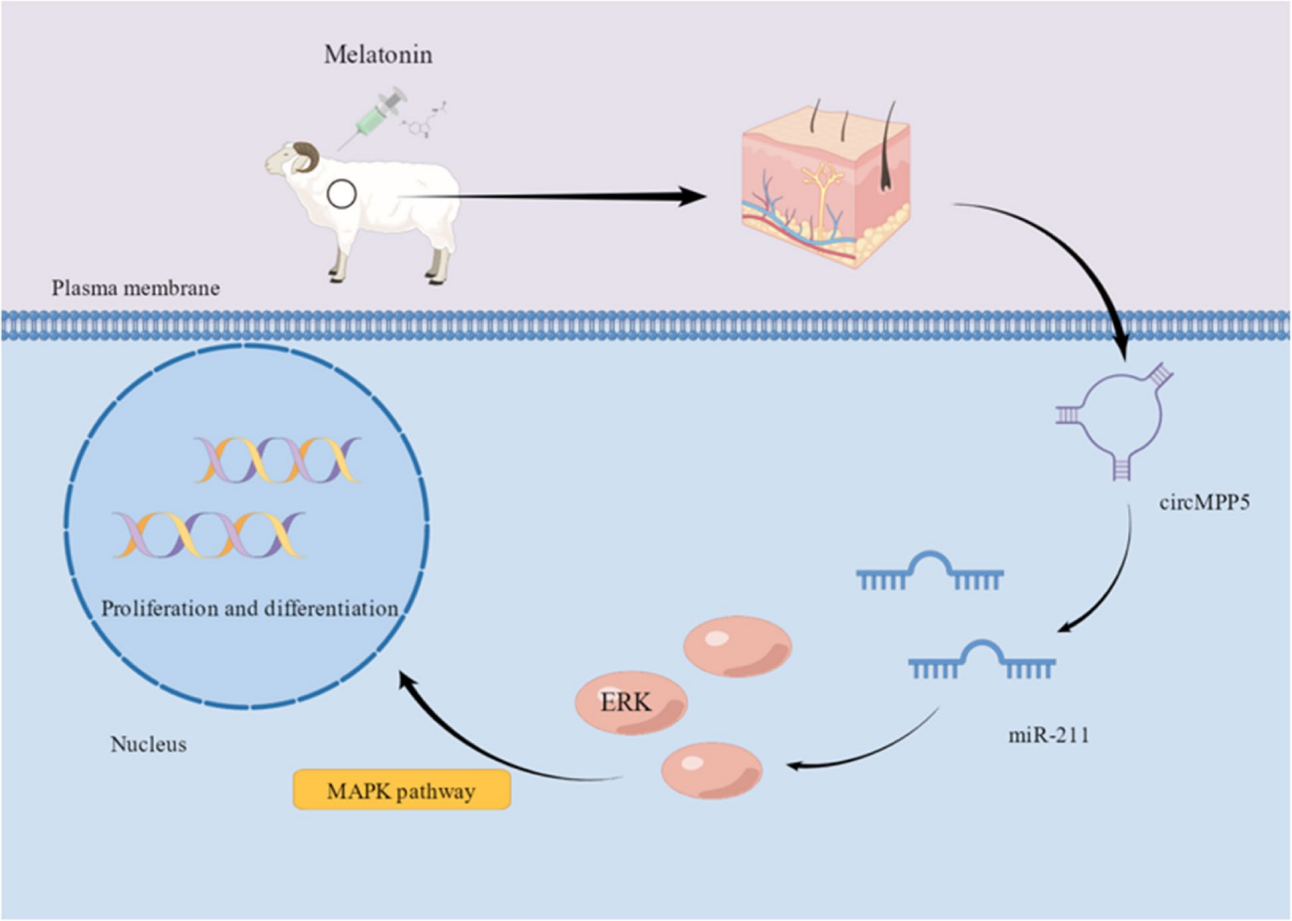
Schematic diagram of the mechanism of the melatonin regulates the mitogen-activated protein kinase (MAPK) pathway via the circMPP5/miR-211/MAPK3 to induce secondary hair follicle differentiation and development in newborn cashmere goat kid

In this study, the results showed that MT can mediate regulation of the MAPK pathway via the circMPP5 sponged the miR-211, regulating the expression of MAPK3, to promote the proliferation and differentiation of secondary hair follicle cells in goat kids (Fig. 13). In conjunction with findings of our earlier studies, results of the current study indicate that MT increases the number of secondary hair follicles in goat kids in two ways: (a) by promoting their differentiation and proliferation, and (b) by reducing the occurrence of apoptosis. The improvement in cashmere fibre quality and yield resulting from treatment of newborn goat kids with MT occurred in the cashmere growth season following insertion of the MT implants and persisted into the following year’s growth period.

## Supplementary Information

**Additional file 1: Table S1.** Nutrient levels of the basal diet (air dry basis) for goat kid. **Table S2.** Summary of identified transcripts. **Table S3.** Differentially expressed mRNA, lncRNA, circRNA and miRNA.

## Data Availability

The datasets used and analyzed during this study are available from the corresponding author upon reasonable request.

## Abbreviations

circRNA: CircularRNA
DAPI: 2-(4-Amidinophenyl)-6-indolecarbamidine dihydrochloride
DE RNA: Different express RNA
ERK: Extracellular regulated protein kinase
FGF: Fibroblast growth factor
FGFR: Fibroblast growth factor receptor
Fn-1: Fibronectin-1
GO: Gene Ontology
K-14: Keratin 14
KEGG: Kyoto Encyclopedia of Genes and Genomes
lncRNA: Longnon-codingRNA
mRNA: MessengerRNA
miRNA: MicroRNA
MT: Melatonin
MAPK: Mitogen-activated protein kinase
PI3K-Akt: Phosphatidylinositol 3-kinase-protein kinase
SD: Standard Deviation
SDS-PAGE: Sodium dodecyl sulfate polyacrylamide gel electrophoresis
WT: Wild type
Wnt-10a: Wingless-type MMTV integration site family, member 10a
