# Supplementary material for: Melatonin promotes the development of the secondary hair follicles by regulating circMPP5
Source: J Anim Sci Biotechnol. 2023 Apr 7;14:51. doi: 10.1186/s40104-023-00849-w (PMC10080870; doi:10.1186/s40104-023-00849-w)
Supplement: Supplementary file 1 — Additional file 1: Table S1. Nutrient levels of the basal diet (air dry basis) for goat kid. Table S2. Summary of identified transcripts. Table S3. Differentially expressed mRNA, lncRNA, circRNA and miRNA. [file 40104_2023_849_MOESM1_ESM.docx]

**Table S1** Nutrient levels of the basal diet (air dry basis) for goat kid

| **Nutrients** | **CP** | **CF** | **Ash** | **Water** | **NaCl** | **Ga** | **Total P** | **Lys** |
| --- | --- | --- | --- | --- | --- | --- | --- | --- |
| Level, % | 20 | 8 | 9 | 14 | 0.5 | 0.5 | 0.4 | 0.5 |

**Table S2** Summary of identified transcripts

| **RNA Type** | **Known** | **Novel** | **Total** |
| --- | --- | --- | --- |
| mRNA | 30,036 | 3201 | 33,237 |
| lncRNA | 2864 | 6996 | 9860 |
| miRNA | 426 | 453 | 879 |
| circRNA | -- | 12,702 | 12,702 |

**Table S3** Differentially expressed mRNA, lncRNA, circRNA and miRNA

| **RNA-Type** | **Regulation** | | **Total** |
| --- | --- | --- | --- |
|  | **Up** | **Down** |  |
| mRNA | 609 | 435 | 1044 |
| lncRNA | 46 | 45 | 91 |
| circRNA | 484 | 570 | 1054 |
| miRNA | 33 | 28 | 61 |
